# Oscillating Laser Welding of TC4-304SS Dissimilar Joints: Regulating Microstructure and Mechanical Properties via Cu, Mo, and Nb Interlayers

**DOI:** 10.3390/ma19010086

**Published:** 2025-12-25

**Authors:** Zhi Cheng, Zheng Wang, Yanfei Bian, Yuanyuan Cheng, Shiming Huang, Chunhuan Chen

**Affiliations:** 1 School of Materials Science and Engineering, Dalian Jiaotong University, Dalian 116028, China; czchzhi@djtu.edu.cn (Z.C.);; 2 Hebei Jinhan Electronic Technology Co., Ltd., Shijiazhuang 065701, China

**Keywords:** titanium-steel dissimilar welding, oscillating laser, interlayer, intermetallic compounds, mechanical properties

## Abstract

This study investigated the use of oscillating laser welding with Cu, Mo, and Nb interlayers to mitigate the issue of brittle intermetallic compound (IMC) formation in titanium-steel dissimilar welding for TC4/304SS lap joints. This study systematically examined the influence of interlayer type on joining mechanisms, microstructure, and mechanical properties. Results indicated that direct welding produced Ti-Fe IMCs (TiFe phase) through fusion, resulting in a thick brittle layer. The Cu interlayer facilitated fusion welding while promoting the formation of TiFe_2_ and TiCu_4_ phases. Mo and Nb interlayers, through fusion-brazing and brazing mechanisms, respectively, inhibited Ti-Fe IMCs by generating Fe-Mo/Nb IMCs and (Ti, Mo/Nb) solid solutions. Mechanical testing indicated that Mo-interlayer joints exhibited the highest shear strength at 1129.53 N, representing a 31% increase relative to direct joining. This was followed by Nb at 1004.4 N, Cu at 871.6 N, and direct welding at 859.13 N. Fracture transitioned from brittle IMC layers in Cu and direct welding to interfaces between interlayers and TC4 in Mo and Nb systems. This study presents Mo/Nb interlayers as the most effective choice for high-strength Ti-steel joints, providing insights for the selection of interlayers in engineering applications.

## 1. Introduction

The ongoing progress in global manufacturing technology has led to the increased application of heterogeneous metal composite structures in diverse fields [1,2]. These structures leverage the principle of complementarity and maximize material utility, thereby effectively harnessing the performance advantages and economic benefits of diverse metals. This underscores their growing significance in modern manufacturing [3,4]. Titanium and its alloys are recognized for their high specific strength, low coefficient of thermal expansion, and low thermal conductivity, leading to widespread applications in aerospace and various other industries [4]. Tuninetti et al. [5] contributed to the structural integrity design and performance optimization of aero-engines by introducing the Johnson–Cook constitutive parameters for titanium alloys and applying them to the simulation of turbofan engine blade containment tests. Stainless steel, frequently utilized as a structural material in engineering, provides superior mechanical properties [6]. Reliable welding between titanium and stainless steel integrates the advantageous properties of both materials while decreasing structural manufacturing costs, thereby improving economic efficiency [7].

The welding of titanium-steel dissimilar metals presents a notable challenge due to the tendency to generate brittle Ti-Fe intermetallic compounds (IMCs) during the process [2]. The presence of these brittle phases significantly diminishes the plasticity and toughness of the joint, resulting in a considerable decrease in strength or potential cracking [8]. Moreover, the distinct variations in physical properties between titanium and steel [9] lead to significant residual welding stresses during the cooling process, potentially causing interfacial cracking.

The main welding techniques for joining titanium alloys to steel include brazing, arc welding [10,11], and laser welding [12]. Brazing [13,14] facilitates metallurgical bonding through melting a filler metal, with its primary advantage being the reduced joining temperature, which effectively inhibits the formation of brittle Ti-Fe IMCs. However, the load-bearing capacity and environmental adaptability of brazed joints are limited by the properties of the filler metal. Arc welding [15], characterized by its cost-effectiveness and ease of operation, is one of the most widely used techniques in modern engineering. Controlling heat input in arc welding presents a challenge: excessive heat input often leads to the significant formation of intermetallic compounds (IMCs). In comparison to the previously discussed methods, laser welding presents notable advantages, including high energy density, focused heat input, and rapid cooling rates, which facilitate improved regulation of the interfacial thermal cycle and IMC formation. Contemporary oscillating laser welding is characterized by increased scanning speeds and a marked reduction in linear energy density. This modulation of the welding thermal cycle through beam oscillation effectively controls IMC formation, resulting in superior joint quality [16].

The extreme brittleness of Ti-Fe IMCs formed during direct titanium-steel joining frequently results in post-weld cracking. Consequently, the incorporation of an interlayer has emerged as a crucial technological strategy to mitigate the challenges associated with titanium-steel welding. The interlayer serves several key functions, including physical isolation, property gradation, and interface regulation [17], which collectively suppress brittle phase formation and alleviate welding stresses. Interlayer materials frequently utilized comprise Cu [18,19], Ni [20], and Nb [21]. X. Hao et al. [22] found that the incorporation of copper foil significantly diminished the solubility of titanium alloy, which in turn reduced the formation of brittle Ti-based IMCs in the weld. This modification mitigated the occurrence of transverse cracks induced by elevated longitudinal stresses in titanium alloy-stainless steel joints. A. Chattopadhyay [23] in situ deposited Ni powder as an interlayer to join titanium and stainless steel dissimilar metals. The results showed that the Ni-interlayer reduced the longitudinal cracks by serving as an effective barrier to elemental diffusion, thereby restricting the growth of brittle intermetallics. As the thermal expansion coefficient of Ni lies between that of stainless steel (SS) and Ti, this acted as a functionally graded layer to reduce the thermal residual stresses, thus preventing the formation of transverse cracks across the joint. The maximum ultimate tensile strength of the weld joints obtained was 375 MPa. Y. Zhang et al. [24] demonstrated that the incorporation of Nb as an interlayer in the laser welding of titanium and steel significantly decreased the propensity for cracking relative to direct welding. This reduction was attributed to the limitation of melting amounts of titanium alloy and stainless steel in the weld pool, which in turn reduces the Ti and Fe content. Research indicates that the incorporation of an interlayer inhibited direct contact between titanium and steel, diminished the formation of Fe-Ti IMCs, improved the stability of interfacial bonding, optimized the microstructure, and significantly enhanced joint strength and plasticity.

This study proposes the application of oscillating laser welding for lap joining of titanium-steel dissimilar metals, where niobium (Nb), molybdenum (Mo), and copper (Cu) interlayers are utilized to promote sound joint formation and enhance interfacial bonding strength. This research systematically examined the formation mechanism of Ti-SS dissimilar metal joints under conditions of laser oscillating welding. The influence of various interlayers on the microstructure and mechanical properties of the joints in the oscillating welding process was studied, and the impact of interlayer type on the microstructure and mechanical properties of Ti-SS dissimilar metal joints was evaluated, offering a theoretical foundation for interlayer selection in future engineering applications.

## 2. Materials and Methods

### 2.1. Materials

The base materials used in this study were 0.5 mm-thick sheets of TC4 titanium alloy (TC4) and 304 stainless steel (304SS), each with dimensions of 50 mm × 100 mm. The chemical compositions are presented in Table 1 and Table 2, respectively. This study examined the influence of various interlayers on the microstructure and properties of titanium-steel joints. As the most widely utilized α + β titanium alloy in the aerospace field, TC4 exhibits a relatively high temperature sensitivity coefficient and a low strain hardening exponent [5]. This characteristic indicates that TC4 is significantly influenced by temperature, and it possesses limited plastic deformation capacity. Pure niobium (Nb), pure molybdenum (Mo), and pure copper (Cu) foils, each with a thickness of 0.2 mm and dimensions of 100 mm × 10 mm, were chosen as interlayers for oscillating laser welding.

### 2.2. Welding Methods

**Figure 1:**
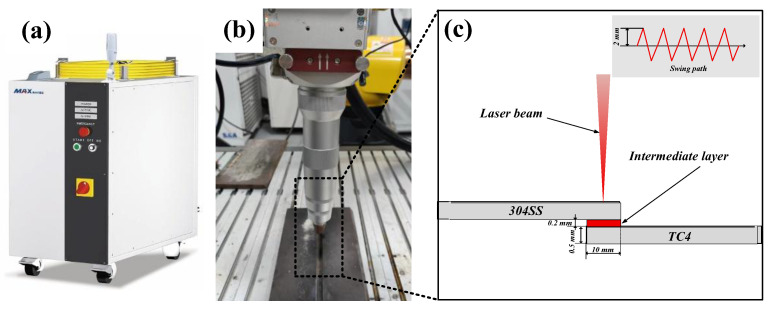
(**a**) MFSC-12000W laser welding equipment; (**b**) laser welding torch; (**c**) titanium-steel joint assembly diagram.

As shown in Figure 1a, a Chuangxin MFSC-12000W laser welding system (manufactured in Shenzhen, China) was employed for the welding experiments. The laser source has a maximum output power of 12 kW, a central wavelength of 1080 nm, and an adjustable oscillation frequency spanning from 0 to 600 Hz. Single-pass welding was conducted using a linear oscillation mode oriented perpendicular to the direction of the weld seam. The lap width of the welded workpieces was established at 10 mm, aligning with the interlayer width. The laser beam was focused on the center of the weld lap area, exhibiting an oscillation width of 4 mm. The welding torch was oriented vertically relative to the workpiece, utilizing 99.999% high-purity argon gas as the shielding gas, with a gas flow rate set at 8 L/min.

Before welding, the faying surfaces of the specimens were ground with sandpaper to remove surface contaminants and oxide layers, thereby ensuring optimal welding conditions. The specimens were arranged in a lap configuration, with the stacking sequence from top to bottom consisting of the 304SS sheet, the interlayer, and the TC4 sheet, as illustrated in Figure 1c. Preliminary experiments indicated that in contrast to direct welding, specimens incorporating interlayers required higher heat input to achieve adequate metallurgical bonding between the sheets. Furthermore, since the melting point of molybdenum (Mo, 2620 °C) is significantly higher than that of copper (Cu, 1083 °C), and Mo exhibits a higher melting point and lower diffusion rate compared to niobium (Nb), the alloying reaction requires a higher temperature. Additionally, the formation energy of intermetallic compounds and the threshold temperature of solid solution are higher, necessitating more heat to drive the interfacial reaction. Therefore, when Mo is employed as the interlayer, the heat input parameters should be further increased to ensure metallurgical bonding of the welded joint and guarantee joint integrity. Parameter selection aims to ensure that various intermediate layers possess optimal forming parameters identified through extensive exploratory experimental conditions during the initial phase, as shown in Table 3. A control group utilizing direct titanium-steel welding was incorporated to examine the regulatory influence of various interlayers on interfacial metallurgy.

**Figure 2:**
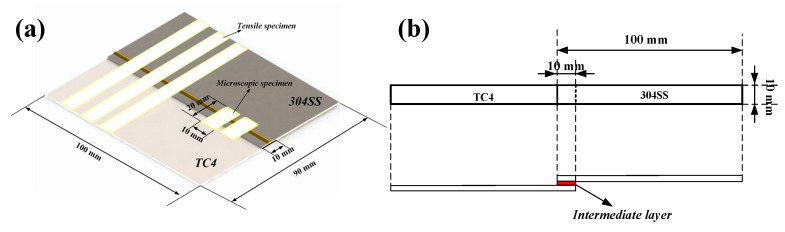
(**a**) Schematic diagram of swing laser welding structure; (**b**) schematic diagram of tensile specimen.

Welded specimens were sectioned using wire electrical discharge machining (EDM) into samples of dimensions 10 mm × 20 mm, as shown in Figure 2a, followed by sequential cleaning with petroleum ether and ethanol, and air-drying. The specimens were ground with 240–3000 grit sandpapers, polished. Subsequently, the samples were rinsed with ethanol a second time prior to the observation of the overall cross-sectional morphology using the Leica DMi8C inverted metallurgical microscope (Leica Microsystems, Shanghai, China). The microstructures of the weld zone were observed using a Carl Zeiss scanning electron microscope (SEM, Oberkochen, Germany), and compositional analysis was performed on the characteristic regions. The experiments were conducted at a high accelerating voltage of 15 kV, with the specimens observed using an ASB detector produced by Fivetrees Company in Shenzhen, China.

Three standard tensile shear specimens were cut, each with dimensions of 10 mm × 90 mm, as illustrated in Figure 2b. The shear force (F_m_) and shear strength (τ) of the welded joints were evaluated at room temperature using a Meters UTM5105 Universal Testing Machine (UTM), manufactured by Meters Company, Jinan, China, with a cross-head speed set at 0.5 mm/min. The results indicate the average of three tests conducted. Microhardness measurements were performed using A Hengyi EA-2A Fully Automatic Microhardness Tester (MHT), manufactured by Hengyi Company, Shanghai, China, applying a load of 25 gf and a duration of 15 s. Measurement points were separated by a distance of 100 μm.

## 3. Results

### 3.1. Joint Surface Appearance and Macrostructure

**Figure 3:**
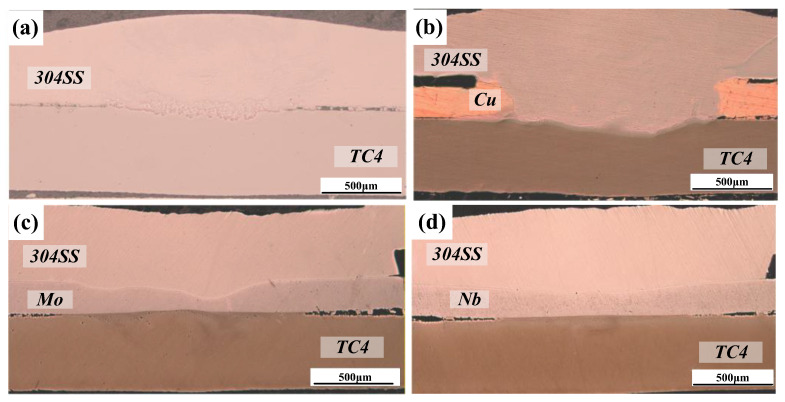
Cross-sectional micrographs of different welded joints under optical microscopy. (**a**) Direct joining; (**b**) Cu interlayer; (**c**) Mo interlayer; (**d**) Nb interlayer.

**Figure 4:**
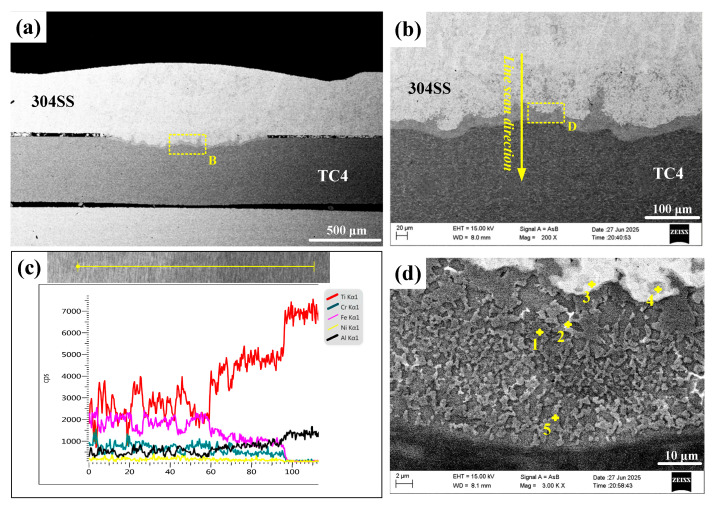
SEM micrographs of direct laser-welded TC4-304SS joints. (**a**) Cross-section of the joint; (**b**) enlarged view of area B in region (**a**) and schematic of the line scan path; (**c**) line scanning results; (**d**) enlarged image of region D in (**b**).

Figure 3 and Figure 4 show the surface appearances and cross-sectional macrostructure of titanium-steel dissimilar metal welded joints with different interlayers. Traditional laser welding is unsuitable for direct joining of Ti-steel dissimilar metals. In contrast, oscillating laser welding, characterized by uniform energy distribution and accurate energy control, has effectively accomplished direct lap joining of Ti-steel dissimilar metals, demonstrating notable advantages in this welding process. The upper surface of the welds produced under oscillating laser conditions exhibited satisfactory formation, maintaining a consistent width of approximately 4 mm, in alignment with the set oscillation width. The surfaces were largely devoid of defects, such as pores, spatter, or cracks. The cross-sectional macrostructure revealed different melting behaviors of the interlayers attributed to their unique physical properties. Characterized by their high melting points, the Nb and Mo interlayers remained partially unmelted during the welding process. The Cu interlayer, characterized by a lower melting point, underwent significant melting, resulting in adequate fusion bonding with the upper 304SS sheet.

### 3.2. Microstructure of Ti-SS Dissimilar Metal Joints

#### 3.2.1. Microstructure of the Directly Welded TC4-304SS Joint

Figure 4 shows the microstructure of the joint obtained through direct laser welding of TC4 and 304SS. The cross-section exhibited no voids or cracks, indicating effective weld formation. Figure 4a shows the overall cross-sectional image, indicating that both the titanium and steel base metals melted to varying extents, which suggests a typical fusion welding mechanism. Figure 4b presents a magnified view of the TC4-304SS interface, revealing a wavy, band-like bonding layer with an approximate thickness of 20 μm. Elemental line scanning across this region, as shown in Figure 4c, confirmed the comprehensive mixing and diffusion of Ti and Fe elements at the interface between TC4 and the IMC layer, indicating sufficient interaction between Fe and Ti. According to the Ti-Fe phase diagram [25], metallurgical bonding was achieved through the formation of Ti-Fe IMCs.

Observation of the IMC layer in the weld zone, as shown in Figure 4d, along with EDS point analysis (Table 4), revealed that this layer mainly comprised a uniformly distributed dark matrix phase (α-Ti + TiFe, points 1, 2, 5) and light-colored granular or short-rod-shaped secondary phases (TiFe, points 3, 4) embedded within the matrix. The existence of a thick IMC transition layer suggests the mutual diffusion of Ti and Fe elements, resulting in bonding through Ti-Fe metallurgical reaction. Under the current conditions, the TiFe_2_ phase was not observed. Research indicates that TiFe_2_ exhibits high brittleness, and its substantial formation would likely lead to immediate post-weld cracking. The suppression of TiFe_2_ formation facilitated the effective direct joining of TC4-304SS through oscillating laser welding.

#### 3.2.2. Microstructures of TC4-304SS Joint with Cu Interlayer

**Figure 5:**
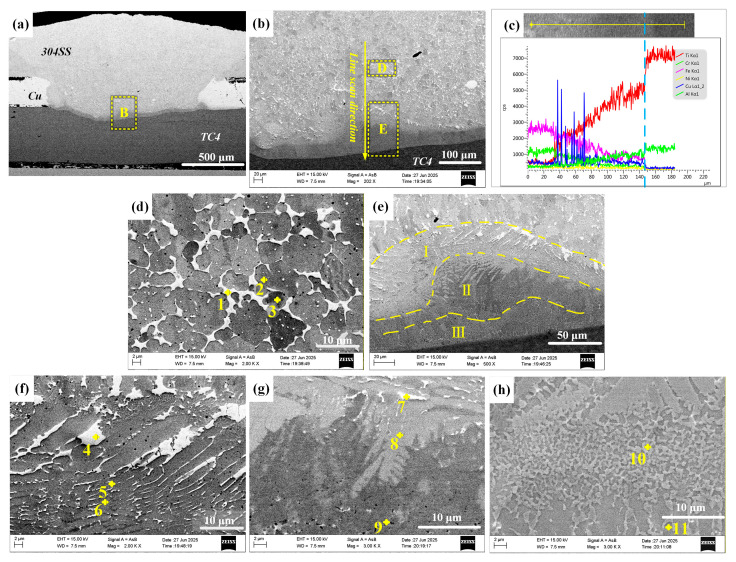
SEM images of Cu-interlayer-assisted TC4/304SS laser-welded joints. (**a**) Cross-section of the joint; (**b**) enlarged view of area B in region (**a**) and schematic of the line scan path; (**c**) line scanning analysis data corresponding to path in (**b**); (**d**) magnified image of area D in (**b**); (**e**) magnified image of area E in (**b**); (**f**) magnified image of area I in (**e**); (**g**) magnified image of area II in (**e**); (**h**) magnified image of area III in (**e**).

The microstructures of the joint formed with a Cu interlayer are shown in Figure 5. Figure 5a shows the joint, which consists of an upper light-colored fusion zone of 304SS and Cu, a Cu/TC4 interface region, and the lower TC4 base metal. The elevated heat input resulted in the complete melting and thorough mixing of 304SS and Cu. The positioning of the TC4 at the bottom, coupled with its high melting point, resulted in only partial melting, which suggested a fusion welding mechanism. A wavy, band-like layer approximately 30 μm thick facilitated bonding at the TC4/Cu interface (Figure 5a). The EDS line scan analysis across the weld, as shown in Figure 5c, showed a progressive reduction in Fe content from top to bottom, coinciding with a corresponding increase in Ti content until reaching the TC4 base metal. This suggests that the Cu interlayer was ineffective in preventing the mutual diffusion of Ti and Fe, as both elements were detected in areas previously occupied by the Cu interlayer.

Magnified observation of area D (Figure 5d) and EDS point analysis (Table 5) revealed that this region was primarily composed of a light gray (Cu, Fe) solid solution (s.s.) phase (point 2), white Cu(s.s.) particles (point 1), and a minor presence of dark Fe(s.s.) phase (point 3). The Cu-Fe binary phase diagram [26], indicates that Cu and Fe have limited mutual solubility and do not form IMCs. Area E (Figure 5e) can be categorized into three sub-layers based on microstructure: Layer I, which is adjacent to the filler metal Cu; Layer II, located in the middle; and Layer III, which is adjacent to TC4 (Figure 5f–h). The line scanning data (Figure 5c) indicate a progressive decrease in Cu and Fe concentrations alongside a rise in Ti content from Layer I to Layer III. Point analysis revealed that Layer I was predominantly a light gray TiFe matrix (point 5), with elongated and blocky white copper solid solution (Cu(s.s.)) phases (point 4) and very fine dark gray TiFe_2_ stringers (point 6) dispersed within it. Layer II consisted of a light grey TiFe phase (point 8), a dark gray TiFe + Ti_2_Cu phase (point 9), and minor white Cu(s.s.) particles. Layer III exhibited an alternating distribution of the light gray TiFe_2_ +TiCu_4_ phase (point 10) and TiFe_2_ + TiCu_4_ phase (point 11). The presence of Ti-Fe intermetallic compounds in all three layers indicated that although the Cu interlayer enabled successful joining under the specified welding parameters, the melting of the interlayer impeded the effective separation of TC4 and 304SS, leading to the formation of brittle Ti-Fe compounds.

#### 3.2.3. Microstructures of TC4-304SS Joint with Mo Interlayer

**Figure 6:**
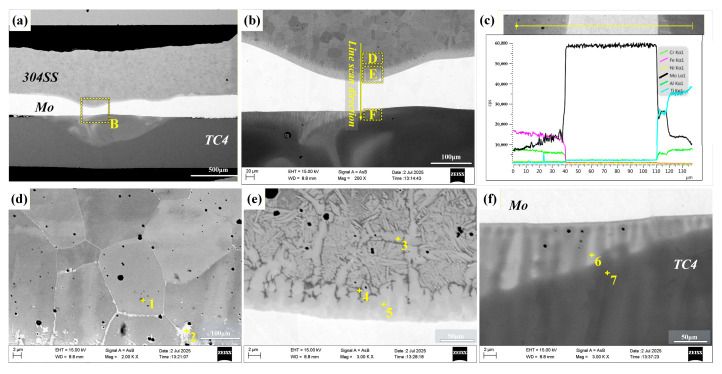
SEM images of Mo-interlayer-assisted TC4/304SS laser-welded joints. (**a**) Cross-section of the joint; (**b**) enlarged view of area B in region (**a**) and schematic of the line scan path; (**c**) line scanning analysis data corresponding to (**b**); (**d**) enlarged image of area D in (**b**); (**e**) enlarged image of area E in (**b**); (**f**) enlarged image of area F in (**b**).

The microstructures of the joint formed with a Mo interlayer are shown in Figure 6a. The joint consists of a 304SS/Mo interface transition region, the Mo interlayer, and a Mo/TC4 interface transition region. Figure 6b shows that the interface between the 304SS base metal and the Mo interlayer experienced melting and considerable interfacial deformation as a result of the high heat input. In contrast, the interface with the TC4 base metal maintained a relatively straight profile. The joint exhibited strong interfacial bonding, free from pores or cracks. Line scanning analysis across the interface, as shown in Figure 6c, revealed significant reductions in Fe and Ti content on both sides of the Mo interlayer, highlighting its effective role as a physical barrier to Ti-Fe interdiffusion. In contrast, the Mo content increased gradually towards the interlayer from both the 304SS and TC4 sides, indicating the diffusion of Mo atoms into both base metals during the welding thermal cycle.

Analysis of EDS points in areas D, E, and F, as shown in Figure 6b, was conducted. The element point scanning results are shown in Table 6. Area D (Figure 6d) exhibited a uniform grain structure consisting of extensive regions of gray-white α-Fe + FeMo phase (point 1), interspersed with the white Fe_3_Mo phase (point 2). Area E (Figure 6e) primarily comprised the dendritic gray-white Fe_3_Mo phase (point 3), with minor quantities of incorporated dark grey α-Fe + FeMo phase (point 4). Area F, located at the interface between Mo and TC4 (Figure 6f), was composed of a gray-white Ti-rich (Ti, Mo)s.s phase (point 6) and a dark gray (Ti, Mo)s.s phase (point 7). The Mo interlayer achieved metallurgical bonding with the upper 304SS through a fusion mechanism and with the lower TC4 titanium alloy via a brazing mechanism, thereby ensuring robust joint strength. The Mo interlayer’s physical barrier effect significantly inhibited the formation of brittle Ti-Fe IMCs, thereby guaranteeing the optimization of joint performance.

#### 3.2.4. Microstructures of TC4-304SS Joint with Nb Interlayer

**Figure 7:**
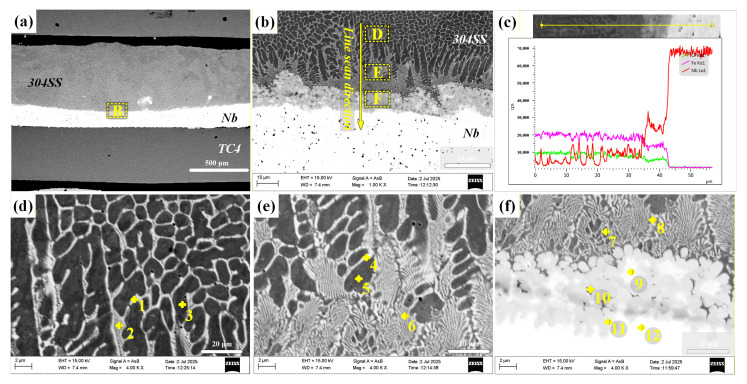
SEM images of Nb-interlayer-assisted TC4/304SS laser-welded joints. (**a**) Overall cross-sectional image of the weld; (**b**) enlarged view of area B in region (**a**) and schematic of the line scan path; (**c**) line scan analysis data corresponding to (**b**); (**d**) enlarged image of area D in (**b**); (**e**) enlarged image of area E in (**b**); (**f**) enlarged image of area F in (**b**).

The microstructures of the joint formed with a Nb interlayer are shown in Figure 7. Figure 7a shows that the joint consisted of a 304SS/Nb interface region, a Nb interlayer region, and a Nb/TC4 interface region. The 304SS interface showed a slight depression, while the TC4 interface remained linear. However, the quality of the joint formation was suboptimal, characterized by a noticeable absence of tight bonding and the presence of numerous defects between the Nb interlayer and the lower TC4 base metal. EDS line scanning analysis along the 304SS/Nb interface, as indicated in Figure 7b, revealed three distinct microstructural zones. The line scanning data, as shown in Figure 7c, indicates abrupt variations in Fe and Nb content at the interface between the upper 304 stainless steel (304SS) and the Nb interlayer. This confirms the mutual diffusion of Fe and Nb, with more pronounced diffusion observed in the light gray region.

An analysis of points in areas D, E, and F, as shown in Figure 7b, was performed. The element point scanning results are shown in Table 7. Area D (Figure 7d) was primarily characterized by a honeycomb-like arrangement of the dark gray α-Fe phase (point 3), accompanied by the minor presence of the light gray Fe_2_Nb + α-Fe phases (points 1, 2). Area E (Figure 7e) comprised a network of gray-white Fe_2_Nb + α-Fe phase (points 4, 6) and a blocky dark gray α-Fe phase (point 5). Area F (Figure 7f) comprised an upper layer characterized by a network of gray-white and dark grey phases, a middle layer consisting of a mixture of dotted light grey Nb_2_Fe phase (point 8) and white Fe_7_Nb_6_ + Nb phase (point 9), and a lower layer with a widespread distribution of white Nb phase (point 12). The phase analysis indicated that bonding between 304SS and the Nb interlayer was achieved through the formation of Fe-Nb IMCs, specifically Fe_2_Nb and Fe_7_Nb_6_.

### 3.3. Mechanical Properties of TC4-304SS Joints with Different Interlayers

**Figure 8:**
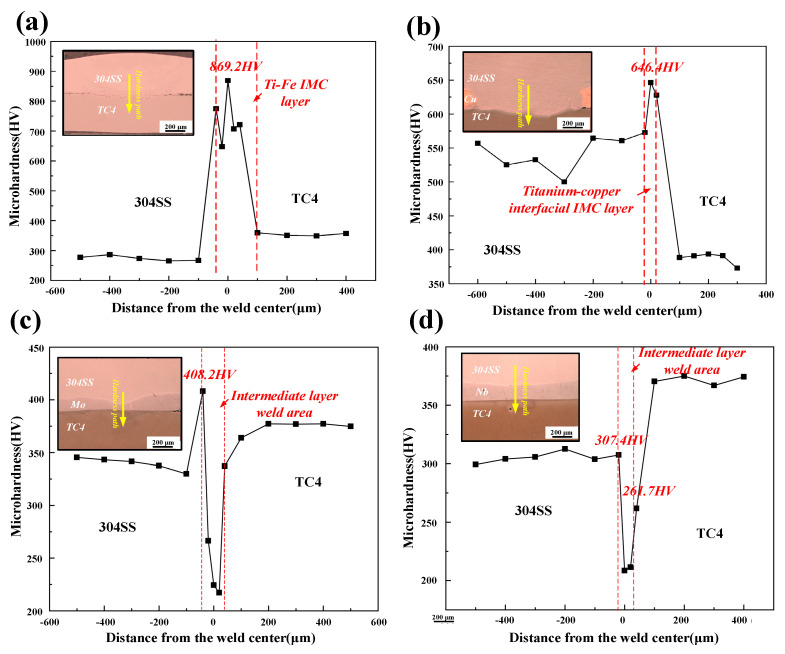
Microhardness images of weld joints with different intermediate layers. (**a**) Microhardness curve of directly joined joints; (**b**) microhardness curve of joints with Cu as the intermediate layer; (**c**) microhardness curve of joints with Mo as the intermediate layer; (**d**) microhardness curve of joints with Nb as the intermediate layer.

The mechanical performance variations among the different joints were assessed through experimental measurements of shear force and micro-Vickers hardness. Figure 8 presents the hardness profiles across the joints from the 304SS side to the TC4 side. The hardness distributions exhibited considerable variability. The hardness in the 304SS base metal area of the joint with the Cu interlayer was significantly greater than that in the corresponding region of joints with other interlayers. The observed increase was attributed to excessive heat input, resulting in the melting and mixing of the Cu interlayer with the 304SS base metal, which facilitated solid solution strengthening and the formation of fine microstructures.

A wavy, band-like Ti-Fe IMC layer, approximately 20 μm thick, formed in the weld zone of the direct-welded joint. The microhardness of this layer attained a peak value of 869.2 HV, substantially surpassing the hardness of the base metals. The joint with the Cu interlayer demonstrated a hardness peak of 646.4 HV. The melted Cu interlayer was ineffective as a physical barrier, resulting in substantial interdiffusion of Ti and Fe elements and the subsequent formation of hard and brittle Ti-Fe IMCs. In contrast, the microhardness in the weld zones of joints incorporating Mo and Nb interlayers was found to be lower than that of the base metals. The limited formation of IMCs in the Fe-Mo series (e.g., Fe_3_Mo, FeMo, Fe_2_Mo) and the Fe-Nb series (e.g., Fe_2_Nb, Fe_7_Nb_6_) accounted for this phenomenon. Nevertheless, the overall hardness in the weld region remained primarily determined by the intrinsic hardness of the corresponding interlayer materials.

**Figure 9:**
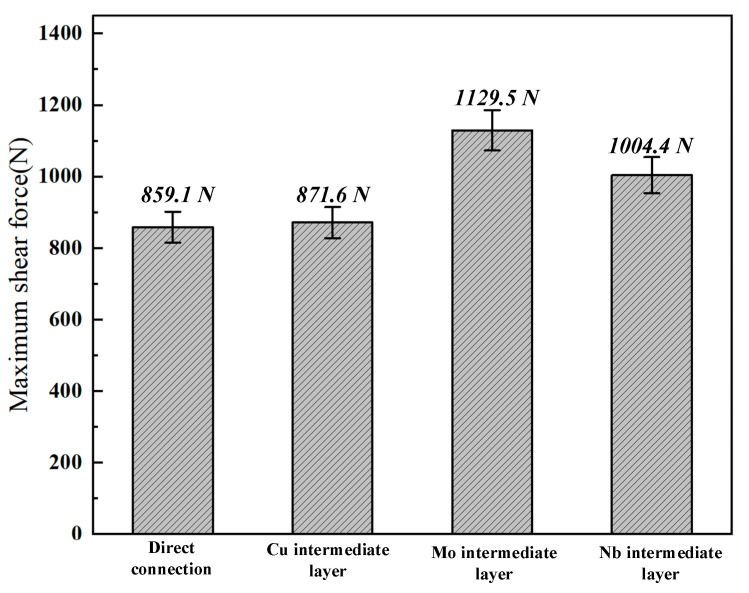
Shear performance of welded joints with different interlayers.

Shear force testing of the tensile specimens was performed at room temperature utilizing an SUS electronic universal testing machine. The shear forces recorded were 859.13 N for the direct joint, 871.6 N for the joint incorporating the Cu interlayer, 1129.53 N for the joint using the Mo interlayer, and 1004.4 N for the joint utilizing the Nb interlayer, as shown in Figure 9. The analysis of microstructural and mechanical properties indicated that the primary cause of fracture in both the direct joint and the joint with the Cu interlayer was the presence of brittle Ti-Fe IMCs. This elucidated the reason for the significantly higher microhardness observed in the weld zones of these two joints compared to the adjacent base metals. In contrast, the joints incorporating Mo and Nb interlayers established connections between the base metals mainly through the formation of Fe-Mo and Fe-Nb IMCs, respectively. This process effectively inhibited the formation of Ti-Fe IMCs, thereby enhancing joint strength.

## 4. Discussion

### 4.1. Joint Formation Mechanisms

#### 4.1.1. The TC4-304SS Joint Without Interlayers

**Figure 10:**
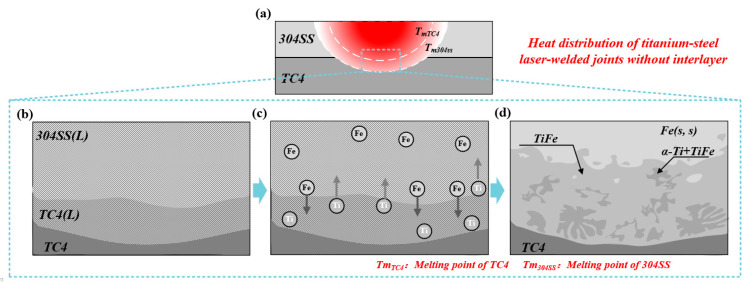
Formation Process of the Direct Titanium-Steel Joint. (**a**) Schematic Diagram of Hypothetical Overall Thermal Distribution Inside the Welded Joint; (**b**) melting and intermixing of Ti and Fe atoms; (**c**) formation sequence of Ti-Fe intermetallic compounds (IMCs); (**d**) phase composition and distribution within the weld zone at room temperature.

Figure 10a illustrates the thermal distribution of the direct joint between TC4 and 304SS dissimilar metals. The oscillating mode from the laser in welding, combined with the heat conduction mode resulting from the current heat input, led to a thermal distribution characterized by a high central temperature and lower surrounding temperatures. Under these conditions, the heat input attained the melting points of 304SS and TC4, resulting in the melting of the base metals. The fusion of Fe and Ti atoms occurred through melting, resulting in the formation of Ti-Fe IMCs, which facilitated a connection via fusion welding. Figure 10 presents a comprehensive analysis of the formation mechanism of the fusion-welded joint. The melting state of the base metals and the formation of compounds during welding can be categorized into two distinct stages.

Stage 1 is illustrated in Figure 10b,c: Under the influence of the laser heat source, the base metals 304SS and TC4 underwent melting. The molten pool at the interface primarily consisted of Fe and Ti atoms, which intermingled in the liquid phase. The precise energy control of the oscillating laser minimized the melting of the Ti-side base metal, thereby effectively suppressing the Ti content in the molten pool.

As shown in Figure 10d, the Ti-Fe binary phase diagram indicates that the primary intermetallic compounds formed were TiFe and TiFe_2_ phases. The high cooling rate associated with laser welding, combined with the localized molten pool exhibiting elevated Fe concentration and reduced Ti concentration, inhibited the formation of the TiFe_2_ phase during the welding process. The joint achieved a connection through the formation of α-Ti and TiFe phases. In comparison to TiFe_2_, the TiFe phase exhibited reduced brittleness and enhanced fracture toughness, thereby mitigating joint stress concentration and decreasing the trend of cracking. Therefore, the primary reason oscillating lasers facilitated direct Ti-steel dissimilar metal connections without cracking was due to their unique operational characteristics.

#### 4.1.2. The TC4-304SS Joint with Cu Interlayers

**Figure 11:**
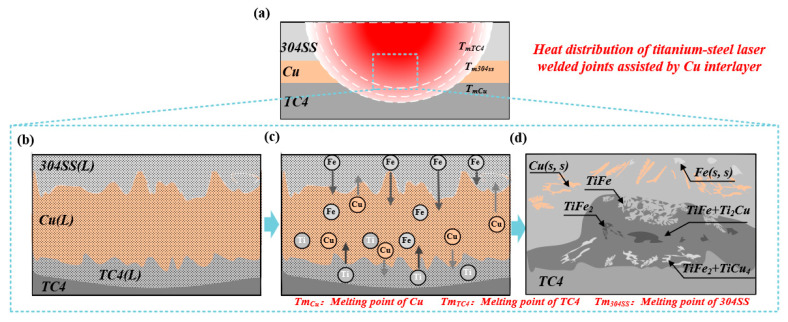
Formation process of titanium-steel dissimilar metal laser welded joints with Cu as the filler metal. (**a**) Schematic Diagram of Hypothetical Overall Thermal Distribution Inside the Welded Joint; (**b**) melting and mixing of Cu, Ti, and Fe atoms; (**c**) formation of Ti-Fe and Ti-Cu intermetallic compound phases; (**d**) phase composition and distribution in the weld zone of the joint at room temperature.

Figure 11a shows the thermal distribution of the laser-welded TC4-304SS dissimilar metal joint utilizing Cu as the interlayer. The existing welding parameters resulted in a maximum welding temperature that attained the melting points of the 304SS base metal, Cu interlayer, and TC4 base metal. The joint attained fusion welding by melting and combining the base metals and interlayer elements. Figure 11 presents a detailed analysis of the joint formation mechanism. The melting and mixing of base metals, formation of intermetallic compounds, and solidification behavior during welding can be classified into two distinct stages based on their temporal sequence.

Stage 1 is depicted in Figure 11b,c: The interface region experienced rapid heating due to the laser heat source. When the temperature surpassed the melting points of the Cu interlayer and base metals, the atoms of Fe, Cu, and Ti underwent significant melting, resulting in the entire region transitioning into a liquid phase. The oscillating laser induced a stirring effect in the molten pool, resulting in complete mixing of Fe, Cu, and Ti atoms in the liquid phase.

Figure 11d illustrates Stage 2. The rapid thermal cycles in laser welding led to a heterogeneous liquid phase composition within the molten pool, characterized by an Fe-rich region at the upper section and a Ti-rich region at the lower section. At a cooling rate that was exceptionally high, the Ti-rich region reached nucleation conditions at 1427 °C, resulting in the precipitation of TiFe_2_ phases from the molten pool in a dendritic morphology with a dispersed distribution. At a temperature of 1085 °C, the liquid phase developed lamellar or rod-like TiFe phases through an eutectic reaction. As the temperature decreased further, the liquid phase in the Cu-rich region experienced a phase transformation, resulting in the formation of Ti_2_Cu phases. These supersaturated Ti_2_Cu phases subsequently precipitated and reacted to produce TiCu_4_ phases, which were distributed throughout the Ti_2_Cu phase. In the joint, Ti-Fe IMCs were generated through the melting of TC4 and 304SS base metals. The melting of Cu mitigates excessive heat input concerns by lowering the local temperature of the molten pool and significantly modifies the thermodynamic equilibrium of the Ti-Cu-Fe ternary system. The incorporation of Cu enhances the solubility range of heterogeneous elements within the system, facilitating the nucleation of binary IMCs such as TiCu/Ti_2_Cu (Ti-Cu) and Cu-Fe, and expediting the precipitation of Ti-Cu-Fe ternary eutectic phases. The oscillating beam effectively regulates the overall heat input. However, the complete melting of the Cu interlayer and the subsequent intense mixing of the molten pool are critical factors that lead to the formation of substantial brittle intermetallic compounds in the joint. However, the dilution effect of Cu prevents the formation of bulk flaky TiFe_2_ phases compared to the joint without an intermediate layer.

#### 4.1.3. The TC4-304SS Joint with Mo Interlayers

**Figure 12:**
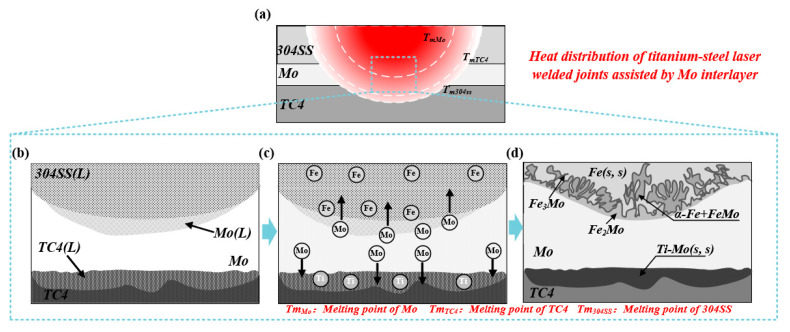
Formation process of titanium-steel dissimilar metal laser welded joints with Mo as the filler metal. (**a**) Schematic Diagram of Hypothetical Overall Thermal Distribution Inside the Welded Joint; (**b**) melting and mixing of Mo, Ti, and Fe atoms; (**c**) formation of Fe-Mo intermetallic compounds and Mo-based solid solution; (**d**) phase composition and distribution in the weld area of the joint at room temperature.

Figure 12a illustrates the thermal distribution of the TC4-304SS dissimilar metal laser-welded joint utilizing Mo as the interlayer. Current welding parameters revealed a temperature gradient: the upper region displayed elevated temperatures, causing the 304SS base metal to reach its melting point and melt. The upper half of the Mo interlayer experienced partial melting upon reaching its melting point, whereas the lower half remained solid as its melting point was not attained. At the Mo/Ti interface, the temperature attained the melting point of TC4, which was lower than that of Mo, resulting in the melting of TC4 while Mo remained in a solid state. Consequently, the interface was bonded through a solid–liquid interface mechanism, and the joint was cohesively formed via a fusion-brazing joining mechanism. Figure 12 presents a detailed analysis of the mechanism involved in the formation of fusion-brazed joints. The formation of the entire region can be summarized into two characteristic stages based on the melting states of the base metals and interlayer, along with the formation of IMCs and solid solutions during welding.

Stage 1 is depicted in Figure 12b,c: The laser heat source caused the melting of the 304SS base metal and the upper portion of the Mo interlayer. The molten pool at the interface consisted mainly of Mo and Fe atoms that intermingled in the liquid phase. Dissolution and diffusion of solid-phase Mo atoms into the liquid-phase TC4 base metal were predominant at the lower Mo/Ti interface.

Figure 12d illustrates Stage 2. The Fe-Mo binary phase diagram indicates the formation of multiple IMCs between Fe and Mo. In contrast, Ti and Mo did not form IMCs and displayed a specific solid solubility in the solid state. At the Fe/Mo interface, the FeMo and Fe_2_Mo phases preferentially nucleated and precipitated, driven by three key factors: low formation free energy, a body-centered cubic (BCC) crystal structure (lattice-matched to the substrate), and low interface energy. As temperature decreased, Mo-lean Fe_2_Mo phases initially precipitated and subsequently reacted with residual Fe atoms in the molten pool to form Fe_3_Mo phases, which exhibited dendritic growth morphology. At the Mo/TC4 interface, atomic interdiffusion resulted in the formation of a (Ti, Mo) solid solution, facilitating the brazing connection of the interface. The upper portion of the Mo interlayer experienced melting; however, its unmelted segment served as an effective physical barrier, completely inhibiting the mixing and reaction between Ti and Fe elements, thus preventing the formation of Ti-Fe IMCs. At the Ti-side interface, a solid solution was formed through a brazing mechanism, while at the steel-side interface, Fe-Mo intermetallic compounds were produced as a result of localized melting.

#### 4.1.4. The TC4-304SS Joint with Nb Interlayers

**Figure 13:**
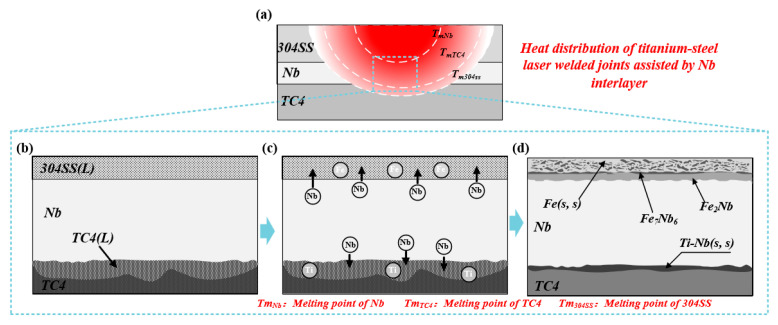
Formation process of titanium-steel dissimilar metal laser welded joints with Nb as the filler metal. (**a**) Schematic Diagram of Hypothetical Overall Thermal Distribution Inside the Welded Joint; (**b**) melting and mixing of Cu, Ti, and Fe atoms; (**c**) formation of Fe-Nb intermetallic compounds and Nb-based solid solution; (**d**) phase composition and distribution in the weld zone of the joint at room temperature.

Figure 13a illustrates the thermal distribution of the TC4-304SS dissimilar metal laser-welded joint utilizing Nb as the interlayer. Under the selected welding parameters, the during the process temperature reached the melting point of the 304SS base metal. At this juncture, the Nb interlayer remained solid, whereas the lower TC4 reached its melting point and liquefied, resulting in an integral connection of the joint through a brazing mechanism. Figure 13 presents a comprehensive analysis of the brazing joint formation mechanism. The melting states of the base metals and interlayer, along with the formation of IMCs and solid solutions during welding, can be categorized into two distinct stages.

Stage 1 is depicted in Figure 13b,c: Under the influence of the laser heat source, both the 304 stainless steel and TC4 base metals underwent melting, whereas the Nb interlayer remained in a solid state. The molten pool at the 304SS/Nb interface consisted mainly of Fe and Nb atoms, where mutual diffusion reactions took place between these atoms at the solid–liquid interface. At the lower Nb/Ti interface, the processes of dissolution and diffusion of Nb atoms into the liquid TC4 phase were predominant.

Figure 13d illustrates Stage 2. The Fe-Nb binary phase diagram indicates the formation of various IMCs between Fe and Nb. In contrast, Ti and Nb did not form IMCs and demonstrated a degree of solid solubility. At the Fe/Nb interface, Fe_2_Nb phases preferentially nucleated and formed as a result of their low formation free energy. As the temperature decreased, certain Fe_2_Nb precipitated and interacted with enriched Nb atoms to produce Fe_7_Nb_6_ phases, which developed in a dendritic morphology. Upon further reduction in temperature, a minor amount of Ti atoms and a substantial quantity of Nb atoms created a (Ti, Nb)s.s, facilitating the brazed joining at the Nb/TC4 interface. The Nb interlayer remained completely unmelted during the process, serving as an effective physical barrier that facilitated brazed joining with the partially melted Ti and SS substrates. The interdiffusion of Ti and Fe elements was impeded, thereby completely suppressing the formation of brittle Ti-Fe IMCs.

### 4.2. Fracture Mechanisms of the Joints

#### 4.2.1. Directly Laser-Welded TC4-304SS Joint

**Figure 14:**
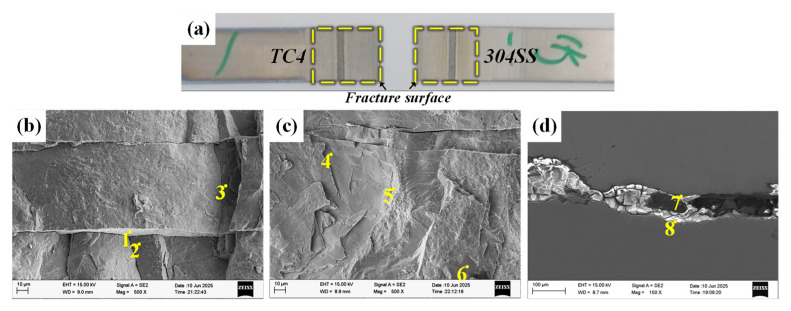
Images of the tensile fracture cross-section of directly connected joints and the fracture path. (**a**) Macroscopic image of the fracture; (**b**) fracture image on the TC4 side; (**c**) fracture image on the 304SS side; (**d**) fracture path.

Figure 14 shows the morphology of the fracture surface and fracture path of the TC4-304SS joint that was welded directly. Analysis revealed that that fracture took place at the weld interface between 304SS and TC4 when subjected to shear loading. The examination of the fracture morphology revealed typical brittle fracture characteristics. The results of EDS point analysis (Table 8) further confirmed that the fracture surface on the TC4 side was primarily made up of the TiFe phase (Points 1, 2), while the fracture surface on the 304SS side was mainly composed of the α-Fe phase and the TiFe_2_ phase (Points 4, 5, 6). The identification of the TiFe_2_ + α-Fe phase (Points 7, 8) on both sides of the fracture path suggests a compromised interface, which was the principal cause of the joint failure.

#### 4.2.2. TC4-304SS Joint with Cu Interlayer

**Figure 15:**
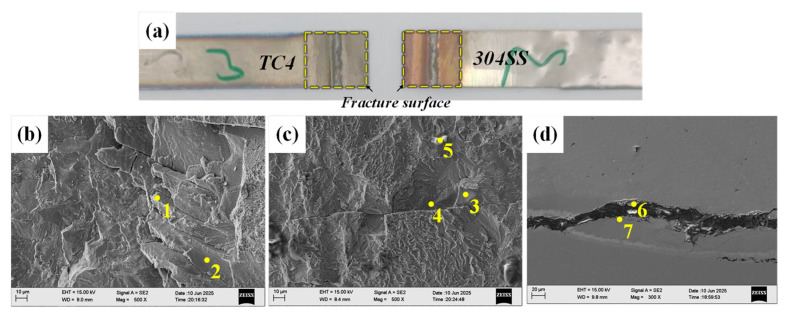
Tensile fracture cross-sectional images and fracture path of the Cu intermediate layer. (**a**) Macroscopic image of the fracture; (**b**) fracture surface on the Ti side; (**c**) fracture surface on the Fe side; (**d**) fracture path.

Figure 15 shows the fracture morphology and the crack propagation path of the joint incorporating the Cu interlayer. Experimental observations indicate that fracture primarily occurred at the welded interface between the Cu interlayer and the TC4 base metal under shear stress. The examination of the fracture surfaces on both the TC4 and Cu sides revealed adequate metallurgical bonding between the Cu interlayer and the TC4 substrate. Combined with previous EDS mapping results, substantial interdiffusion was observed between the Cu interlayer and the 304SS base metal, facilitating the migration of Fe atoms to the Cu/TC4 interface region. The results of EDS point analysis (Table 9) showed the presence of distinct Ti-Fe IMC phases in the interfacial region, while the fracture morphology exhibited typical brittle characteristics. The fracture surface of TC4/Cu was mainly composed of the TiFe_2_ + TiCu_4_ phase (Point 1) and the TiFe phase (Point 2). The fracture surface of 304SS/Cu was predominantly composed of the TiFe phase and Ti_2_Cu phase (Point 3). The identification of the TiFe phase (Points 6, 7) on either side of the fracture path confirms that the fracture primarily took place within the brittle TiFe IMC region.

#### 4.2.3. TC4-304SS Joint with Mo Interlayer

**Figure 16:**
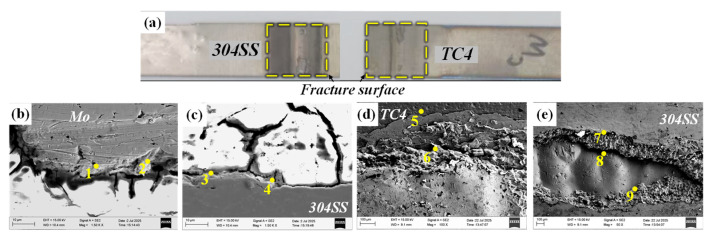
Tensile fracture cross-sectional images and fracture path of the joint with Mo intermediate layer. (**a**) Macroscopic image of the fracture sample; (**b**) fracture path; (**c**) fracture path; (**d**) fracture surface on the Ti side; (**e**) fracture surface on the Fe side;.

Figure 16 shows the fracture morphology and the crack propagation path of the joint incorporating the Mo interlayer. Experimental findings indicate that fractures predominantly occurred at the interface between the Mo interlayer and the TC4 substrate under shear loading. Analysis of fracture morphology shows that the Mo interlayer achieved adequate metallurgical bonding with both 304SS and TC4. Due to the positioning of the 304SS on top during welding, it experienced increased heat input, leading to a higher degree of fusion at the 304SS/Mo interface. The EDS point analysis of the fracture surface (Table 10) showed no significant enrichment of Ti or Fe elements, suggesting the lack of substantial Ti-Fe IMCs at the interface. The fracture surface of TC4/Mo was primarily made up of the (Mo, Ti) solid solution phase (Point 5) and Mo (Point 6). The fracture surface of 304SS/Mo primarily comprised Mo (Points 7, 9) and the Fe_2_Mo phase (Point 8). The presence of both Mo and Ti phases adjacent to the fracture path indicates that the bonding between the Mo interlayer and the lower TC4 base metal was suboptimal, leading to fracture at the TC4/Mo interface.

#### 4.2.4. TC4-304SS Joint with Nb Interlayer

**Figure 17:**
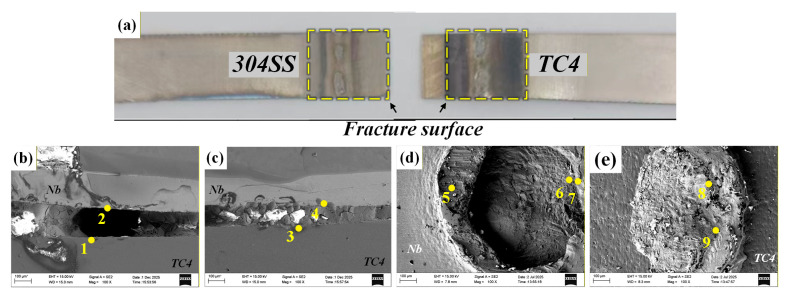
Tensile fracture cross-sectional images and fracture paths of Nb intermediate layer joints. (**a**) Macroscopic fracture image; (**b**) fracture path; (**c**) fracture path; (**d**) fracture surface on the 304SS/Nb side; (**e**) fracture surface on the TC4/Nb side.

Figure 17 shows the fracture morphology and crack propagation path of the joint incorporating the Nb interlayer. Experimental findings indicated that fractures primarily occurred at the interface between the Nb interlayer and the TC4 substrate under shear loading. Examination of the fracture surfaces on the TC4 side (Figure 17e) and the 304SS side (Figure 17d) showed that the Nb interlayer formed adequate metallurgical bonding with the 304 stainless steel. The EDS point analysis of the fracture surface (Table 11) revealed no significant enrichment of Ti or Fe elements, thereby confirming that the physical barrier effect of the Nb interlayer inhibited the formation of substantial Ti-Fe IMCs in the weld. The fracture surface on the 304SS/Nb side primarily consisted of the Fe_7_Nb_6_ + Nb phase (Point 8) and the Fe_2_Nb + α-Fe phase (Point 9). The fracture surface on the Ti/Nb side was primarily characterized by protruding Nb-based solid solution (Point 5) and recessed Fe_7_Nb_6_ + Fe_2_Nb phase (Point 6). Phase composition analysis via point scanning along the fracture path revealed that the fracture surface was uniformly composed of a Nb-based solid solution. Furthermore, microscopic observations clearly indicated insufficient bonding between the Nb interlayer and the underlying TC4 base material (Figure 7a), which was identified as the primary cause of joint failure.

## 5. Conclusions

This research systematically investigated the effects of Cu, Mo, and Nb interlayers on the formation mechanism, microstructure, and mechanical properties of TC4/304SS dissimilar metal joints fabricated through oscillating laser welding. The principal findings are summarized as follows:

1. Direct welding of Ti-SS achieved fusion bonding through Ti-Fe IMCs, predominantly the TiFe phase, with a thickness of approximately 20 μm. The Cu interlayer facilitated fusion welding via Ti-Fe-Cu ternary mixing. The formation of TiFe2 and TiCu4 phases at the TC4 side interface resulted in localized brittleness. Mo and Nb interlayers enabled hybrid bonding mechanisms due to their elevated melting points. The Mo interlayer promoted fusion-brazing through Fe-Mo IMCs in the upper area and (Ti, Mo)s.s in the lower region. Nb interlayer enabled brazing via Fe-Nb IMCs and (Ti, Nb)s.s.
2. The introduction of Mo and Nb interlayers removed the continuous Ti-Fe IMC layers. This modification led to a more uniform hardness distribution (≤400 HV), whereas Cu-interlayer joints exhibited a maximum hardness of 869.2 HV. A moderately lower hardness is beneficial for promoting deformation coordination at the joint, thus improving its mechanical strength.
3. The Mo-interlayer joint demonstrated the highest shear strength at 1129.53 N, followed by Nb at 1004.4 N, Cu at 871.6 N, and direct welding at 859.13 N. Fracture analysis indicated brittle fracture at the interlayer–TC4 interface for Mo/Nb joints. In contrast Cu and direct-welded joints exhibited brittle behavior at Ti-Fe IMCs.
4. Mo and Nb interlayers demonstrated enhanced performance in oscillating laser welding of TC4-304SS dissimilar metals by reducing the formation of brittle IMCs and improving joint strength.

This study presents a theoretical framework for selecting interlayers in engineering applications requiring high-performance titanium-steel joints. However, it is confined to a comparison of the microstructure and mechanical properties of joints with various interlayers under consistent heat input conditions. Future research must investigate the impact of welding parameters on the microstructure of joints with different interlayers and optimize these parameters to improve joint performance.

## Figures and Tables

**Table 1:** Chemical composition of TC4 titanium alloy (wt.%).

Element	Al	V	C	Fe	N	H	O	Ti
Content	5.5–6.8	3.5–4.5	0.1	0.3	0.05	0.01	0.2	Bal.

**Table 2:** Chemical composition of 304 stainless steel (wt.%).

Element	C	Cr	Ni	Mn	Si	S	P	Fe
Content	0.08	19.93	7.71	1.45	0.62	0.014	0.032	Bal.

**Table 3:** Laser welding parameters used for different interlayers.

Intermediate Layer Material	Laser Power (kW)	Welding Speed (mm/s)	Oscillating Frequency (Hz)	Oscillating Width (mm)
No intermediate layer	2.4	30	50	2
Mo	3	28	300	2
Cu	2.76	28	300	2
Nb	2.76	28	300	2

**Table 4:** Phase composition analysis of the directly laser-welded TC4-304SS joint (at.%).

Number	Al	Ti	Cr	Fe	Ni	Possible Phases
1	8.61	64.10	6.47	18.94	1.88	α-Ti + TiFe
2	7.59	65.09	4.55	18.54	4.23	α-Ti + TiFe
3	5.66	42.65	13.63	35.75	2.31	TiFe
4	5.99	45.94	14.09	31.98	2.00	TiFe
5	8.64	64.42	5.41	19.18	2.35	α-Ti + TiFe

**Table 5:** Phase composition analysis of the TC4-304SS joint with Cu interlayer (at.%).

Number	Ti	Cr	Fe	Ni	Cu	Possible Phases
1	0.97	5.31	18.30	3.56	71.15	Cu(s.s.)
2	1.68	14.29	47.94	4.44	31.22	(Fe, Cu)s.s
3	2.61	16.74	62.00	7.59	10.56	Fe(s.s.)
4	4.85	2.57	9.53	4.86	78.19	Cu(s.s.)
5	34.18	7.39	32.27	10.35	15.82	TiFe
6	26.91	14.45	52.71	3.67	2.26	TiFe_2_
7	27.42	8.43	31.00	2.72	8.21	TiFe
8	38.85	12.85	42.57	2.38	3.55	TiFe
9	55.32	4.98	22.97	3.87	12.85	TiFe + Ti_2_Cu
10	68.36	2.67	17.30	3.43	8.24	TiFe_2_ + TiCu_4_
11	73.27	2.52	14.86	2.46	6.44	TiFe_2_ + TiCu_4_

**Table 6:** Phase composition analysis of the TC4/304SS joint with Mo interlayer (at.%).

Number	Ti	Cr	Fe	Ni	Mo	Possible Phases
1	0.36	18.56	65.46	7.01	8.60	α-Fe + FeMo
2	0.71	18.39	58.34	6.31	16.27	Fe_3_Mo
3	0.49	16.35	56.98	6.33	19.86	Fe_3_Mo
4	0.35	14.44	64.81	7.78	12.62	α-Fe + FeMo
5	-	11.42	57.26	3.52	27.80	Fe_2_Mo
6	63.04	1.00	0.09	-	29.15	(Ti, Mo)s.s
7	73.62	1.16	0.10	-	16.28	(Ti, Mo)s.s

**Table 7:** Phase composition analysis of the TC4-304SS joint with Nb interlayer (at.%).

Number	Cr	Fe	Ni	Nb	Possible Phases
1	17.01	61.73	7.67	13.59	Fe_2_Nb + α-Fe
2	17.10	63.44	6.79	12.67	Fe_2_Nb + α-Fe
3	20.68	71.33	6.93	1.06	α-Fe
4	18.02	64.81	7.24	9.93	Fe_2_Nb + α-Fe
5	20.75	70.50	7.49	1.26	α-Fe
6	17.97	64.94	6.89	10.20	Fe_2_Nb + α-Fe
7	20.45	69.74	6.79	3.02	α-Fe
8	16.60	62.09	7.84	13.47	Fe_2_Nb + α-Fe
9	8.19	37.70	5.05	49.06	Fe_7_Nb_6_ + Nb
10	15.06	56.32	5.32	23.31	Fe_2_Nb
11	2.42	7.22	0.69	89.67	Fe_7_Nb_6_ + Nb
12	0.20	0.74	0.07	98.99	Nb

**Table 8:** Phase composition analysis of the fracture surface of the direct joint (at.%).

Number	Al	Ti	Cr	Fe
1	5.00	40.57	12.10	38.78
2	3.62	47.17	12.69	36.53
3	10.89	87.23	0.54	1.34
4	9.69	14.55	6.06	69.69
5	2.32	11.55	20.33	65.80
6	2.29	21.29	16.95	59.46
7	2.30	22.65	-	68.21
8	-	33.46	-	66.54

**Table 9:** Phase composition analysis of the fracture surface of the joint with Cu interlayer (at.%).

Number	Al	Ti	Fe	Cu
1	3.33	31.09	54.14	11.43
2	5.97	50.45	40.10	3.49
3	9.37	58.67	17.25	14.71
4	3.22	76.45	13.89	6.43
5	18.48	67.00	14.52	0.00
6	6.20	45.59	42.48	5.74
7	5.40	44.14	46.92	3.54

**Table 10:** Phase composition analysis of the fracture surface of the joint with Mo interlayer (at.%).

Number	Ni	Ti	Cr	Fe	Mo	Al
1	0.07	0.00	0.07	0.44	99.43	-
2	0.29	1.53	0.02	0.43	97.74	-
3	0.05	98.89	0.41	-	0.65	-
4	0.07	97.94	0.53	-	1.46	-
5	0.00	69.77	0.00	0.11	22.16	6.85
6	0.50	0.21	0.02	0.97	97.88	0.43
7	0.46	-	0.00	1.87	97.10	0.57
8	5.11	-	16.37	53.21	25.17	0.14
9	0.00	-	0.20	1.38	98.33	0.09

**Table 11:** Phase composition analysis of the fracture surface of the joint with Nb interlayer (at.%).

Number	Ti	Cr	Fe	Ni	Nb
1	77.49	5.84	1.23	2.81	18.47
2	0.00	0.00	0.16	0.00	99.83
3	80.37	3.74	0.37	4.86	10.66
4	3.45	8.88	0.28	2.23	85.16
5	16.57	1.54	5.04	6.23	70.62
6	-	15.50	52.92	5.39	14.89
7	0.00	0.04	0.44	0.10	99.42
8	0.00	8.46	33.60	4.56	53.26
9	0.07	13.89	51.13	4.85	30.06

## Data Availability

The data presented in this study are available on request from the corresponding author due to technical or time limitations.
